# Pre-hospital Assessment of Large Vessel Occlusion Strokes: Implications for Modeling and Planning Stroke Systems of Care

**DOI:** 10.3389/fneur.2019.00955

**Published:** 2019-09-13

**Authors:** Fabricio O. Lima, Francisco José Arruda Mont'Alverne, Diego Bandeira, Raul G. Nogueira

**Affiliations:** ^1^Post-Graduate Program in Medical Sciences, Universidade de Fortaleza, Fortaleza, Brazil; ^2^Neurology Service, Hospital Geral de Fortaleza, Fortaleza, Brazil; ^3^Interventional Radiology Service, Hospital Geral de Fortaleza, Fortaleza, Brazil; ^4^Department of Neurology, Marcus Stroke and Neuroscience Center, Grady Memorial Hospital, Emory University School of Medicine, Atlanta, GA, United States

**Keywords:** stroke, large vessel occlusion, pre-hospital assessment, stroke triage, stroke systems of care

## Abstract

The social and financial burden of stroke is remarkable. Stroke is a leading cause of death and long-term disability worldwide. For several years, intravenous recombinant tissue plasminogen activator (IV rt-PA) remained as the only proven therapy for acute ischemic stroke. However, its benefit is hampered by a narrow therapeutic window and limited efficacy for large vessel occlusion (LVO) strokes. Recent trials of endovascular therapy (EVT) for LVO strokes have demonstrated improved patient outcomes when compared to treatment with medical treatment alone (with or without IV rt-PA). Thus, EVT has become a critical component of stroke care. As in IV rt-PA, time to treatment is a crucial factor with high impact on outcomes. Unlike IV rt-PA, EVT is only available at a limited number of centers. Considering the time sensitive benefit of reperfusion therapies of acute ischemic stroke, costs and logistics associated, it is recommended that regional systems of acute stroke care should be developed. These should include rapid identification of suspected stroke, centers that provide initial emergency care, including administration of IV rt-PA, and centers capable of performing endovascular stroke treatment with comprehensive periprocedural care to which rapid transport can be arranged when appropriate. In the pre-hospital setting, the development of scales easier and quicker to perform than the NIHSS yet with a maintained accuracy for detecting LVO strokes is of paramount importance. Several scales have been developed. On the other hand, the decision whether to transport to a primary stroke center (PSC) or to a comprehensive stroke center (CSC) is complex and far beyond the simple diagnosis of a LVO. Ongoing studies will provide important answers to the best transfer strategy for acute stroke patients. At the same time, the development of new technologies to aid in real time the decision-making process will simplify the logistics of regional systems for acute stroke care and, likely improve patients' outcomes through tailored selection of the most appropriate recanalization strategy and destination center.

## Introduction

The social and financial burden of stroke is remarkable. Stroke is the second leading cause of death and the first cause of long-term disability worldwide (1). Approximately 795,000 people experience a new or recurrent stroke in the United States each year and 40% are left with permanent disability (1, 2). Access to reperfusion therapies has changed the landscape of stroke care with dramatic improvements in functional outcomes as measured by the modified Rankin scale (mRS). However, the benefits of both intravenous recombinant type plasminogen activator (IV rt-PA) and endovascular therapy (EVT) are strongly time dependent.

At the pre-hospital setting, the primary goal for emergency medical services (EMS) is to ensure that stroke patients receive the fastest and most appropriate triage in order to optimize their chances of receiving reperfusion treatment. Nonetheless, EVT, a more effective treatment for large vessel occlusion (LVO) than IV rt-PA, is available only in a few centers. The contrasting efficacy for different types of ischemic strokes and the imbalance of the availability of treatments lead to a complex decision where time to treatment has to be weighed against effectiveness in any given clinical scenario.

In this article, we will discuss different aspects involved in the complex decision-making process for pre-hospital assessment and triage of stroke patients as well as current trends that will probably impact future directions in the field.

## The Evolving Field of Reperfusion Therapies in Acute Ischemic Stroke

For almost 20 years, IV rt-PA remained as the only proven therapy for acute ischemic stroke. In the NINDS rt-PA trial, the number needed to treat (NNT) for one additional 90-day excellent functional outcome (mRS ≤ 1) with IV rt-PA in the ≤ 3-h-window was 8.4 (3). In the ECASS-3 trial, the NNT with IV rt-PA in the 3–4.5-h-window for one additional 90-day excellent functional outcome was 20 (4). While IV rt-PA remains a level-IA treatment for acute ischemic stroke, it was early recognized its limited efficacy for LVO (5). In a recent published prospective study using CTA (median time of 132 min from the start of IV thrombolysis to reassessment), the frequency of recanalization of intracranial-ICA, proximal MCA-M1, distal MCA-M1, and MCA-M2 occlusions after IV t-PA was 18.1, 24.2, 17.9, and 32%, respectively (6).

Recent trials of EVT for LVO strokes have demonstrated improved patient outcomes when compared to medical therapy alone (with or without IV rt-PA). The development of endovascular approaches with stent-retrievers and/or thromboaspiration devices have made significant improvements in the treatment of LVO strokes with an average frequency of recanalization of 71% and major functional improvements including those patients with contra-indications for IV rt-PA (7, 8). The frequency of functional independence at 90 days (mRS ≤ 2) ranged from 32.6% up to 71% probably reflecting the type of imaging selection of patients and increased recanalization rates (9–13). As such, EVT has become an essential component of stroke care (5).

The progression of the penumbra to irreversible ischemia is not uniform across patients. Approximately 20–30% patients with LVO stroke fall within the spectrum of ultrafast progressors who may be at particular risk to develop accelerated ischemia with a malignant profile. However, a small proportion of patients (≤30%) are “slow progressors” and can sustain adequate brain perfusion through an extended period allowing for reperfusion at later time windows with sustained benefits (14). A new milestone in the treatment of acute ischemic stroke was the extension of the therapeutic window for EVT up to 24 h in carefully selected patients who have LVO and salvageable brain, defined either by imaging mismatch (diffusion and/or perfusion techniques to evaluate ischemic core and penumbra on MR or CT) or by clinical-imaging mismatch with comparable treatment effects as earlier time windows. Even though patients were treated in an extended time window, the NNT for achieving functional independency at 90 days was 2.8 (absolute difference in the utility weighted mRS 2.1, 95% CI [1.2–3.1]) and 3.6 (OR 2.77; 95% CI [1.63–4.70]) in the DAWN and DEFUSE-III trials, respectively (15, 16).

After several failed attempts, intravenous thrombolysis has also recently experienced a stretch in its therapeutic time window. Based on FLAIR-DWI mismatch the MRI-Guided Thrombolysis for Stroke with Unknown Time of Onset (WAKE-UP) trial showed benefit (mRS ≤ 1 at 90 days) of IV rt-PA for patients with more than 4.5 h from last seen well (OR 1.6, 95% CI [1.1–2.4]; *p* = 0.02) with an acceptable safety profile (17). The EXTEND trial used perfusion imaging to select patients for IV thrombolysis up to 9 h from stroke onset. Patients assigned to treatment were more likely to achieve excellent functional outcome at 3 months (adjusted risk ratio, 1.44; 95% CI [1.01–2.06]; *P* = 0.04) with a rate of symptomatic ICH similar to trials of earlier time windows (adjusted RR, 7.22; 95% CI [0.97–53.54]; *P* = 0.05) (18).

The development of new thrombolytic agents has also contributed to lighten the path of reperfusion therapy in acute ischemic stroke. Tenecteplase (TNK), a modified plasminogen activator with higher fibrin specificity with superior pharmacodynamic and pharmacokinetic properties, was recently compared with alteplase showing improved functional outcomes and higher recanalization rates when compared to IV rt-PA prior to EVT (19). Its efficacy, lower cost, and the simplified logistics associated with a single bolus dose of TNK will likely play an important role in the acute stroke treatment. These advantages make TNK an interesting candidate for use in mobile stroke units which have consistently shown to reduce delays in treatment (20–22).

The recent advances in reperfusion therapy have allowed for an expansion of its benefit to a larger population of patients. However, time to treatment remains a crucial factor with high impact on outcomes in both treatment modalities. The frequency of good outcome after IV rt-PA treatment decreases rapidly with time. The NNT to achieve excellent functional outcome (mRS 0–1) at 90 days rises from 4.5 to 9 and 14.1 for those patients treated within 90, 91–180, and 181–270 min, respectively (23). The same reasoning can be applied to EVT in broadly selected patients with frequencies of good functional outcome (mRS 0–2) ranging from 64.1% for those reperfused within 180 min to 46.1% for those reperfused within 480 min (24).

Some patients may still have mismatch between the amount of critically hypoperfused and infarcted brain at the extended window and may therefore benefit from reperfusion at later times (as shown in DAWN and DEFUSE 3 trials). However, the chances of having a significant mismatch decay over time. As such, the importance of time remains as one of the pillars of stroke emergency care together with effective reperfusion.

## Triage of Stroke Patients in the Pre-hospital Setting

Considering the time sensitive benefit of reperfusion therapies of acute ischemic stroke as well as the costs and complex logistics associated, it is highly recommended that regional systems of acute stroke care should be developed. These should include centers that provide initial emergency care, including administration of IV rt-PA, and centers capable of performing EVT with comprehensive periprocedural care to which rapid transport can be arranged as appropriate (5).

For pre-hospital patients with suspected LVO by a stroke severity scale, the decision whether to transport first to a primary stroke center (PSC) and then to a comprehensive stroke center (CSC)—drip-and-ship model (DS)—or directly to a CSC—mothership model (MS)—is multifactorial. A critical factor is to define whether, by the time the patient gets to the closest PSC vs. CSC, he/she will be a candidate to IV rt-PA only, EVT only, both, or neither one. It thus becomes essential to understand the untoward consequences of suboptimal triage as (1) bypassing the closest PSC to a CSC may (a) decrease the chances of the patient receiving IV rt-PA, (b) prolong the time to IV rt-PA treatment, and (c) unnecessarily saturate the CSC bed capacity with patients that could be well-cared for at a closer PSC, and (2) going to the closest PSC may prolong the time to EVT or even completely preclude its performance.

One variable to be considered is time from stroke onset (or time last seen well if the ictus was unwitnessed or upon awakening). As previously mentioned, time is a strong determinant for the IV rt-PA therapeutic response in stroke. Indeed, the first hour after stroke onset, as in trauma care, has been coined as the “golden hour” for reperfusion therapies and is associated with a higher proportion of patients with favorable and excellent outcome in all age groups (8, 25). As a consequence, if a patient has the possibility to be treated within 1 h of onset, this is a variable that should be taken into account and the patient should be transported to the nearest rt-PA-capable hospital. Having said that, one must also consider any potential rt-PA exclusion criteria in the decision algorithm for the best primary destination stroke center. Moreover, no specific data is available regarding the effect of IV rt-PA during the golden hour in LVO patients, so its impact on the line of care is yet to be established.

Mixed results have been reported in the literature. Some studies have shown added benefit of early IV thrombolysis before thrombectomy in LVO stroke patients in terms of recanalization and functional outcomes (19, 26). Nevertheless, a recent systematic review favors the MS model over the DS for patients with suspected LVO. Those patients that were primarily direct to a CSC (MS model) had significantly better outcomes than patients that were first directed to a PSC and then transferred to a CSC (DS model) (90-day mRS 0–2: 60.0% vs. 52.2%; OR, 1.38; 95% CI [1.06–1.79]; *p* = 0.02). Even though, no difference was found between the treatment pathways in successful reperfusion, patients undergoing MS had better functional outcome than those undergoing DS, probably as a consequence of quicker transfer times (that could go up to 100 min in the DS model) and shorter time-to-reperfusion, known to be a powerful predictor of good outcome (27). However, these are retrospective studies and the results must be interpreted with caution.

Likewise, during inter-hospital transfer, one out of three patients with stroke from anterior circulation LVO becomes ineligible for mechanical thrombectomy (MT) due to progression of ischemic core based on CT ASPECTS imaging criteria (28). This highlights the critical importance of better field triage and rapid transfer for patients with LVO profile to hospitals capable of carrying out MT. Therefore, it is of paramount importance the expeditious transfer process for patients with LVO, which are primarily admitted to a PSC.

The time from PSC arrival to PSC departure (e.g., door in door out; DIDO) has become just as critical of a metric as the door-to-needle (DTN) time. Implementation of organized protocol for interhospital transfer of patients should be established and approved in PSCs, since it is associated with a reduction of PSC DIDO times arrival to the CSC and probably faster reperfusion. One example of such a protocol led to shorter DIDO time (64 vs. 104 min) which explained much of the reperfusion time (132 vs. 179 min). As consequence, patients were twice as likely to have a favorable outcome (OR 2.99 [95% CI, 1.0–8.7]; *p* = 0.04) (29).

Another point to be considered, is the transport times to a PSC or to a CSC as well from the PSC to the CSC. The decision about the most suitable type of transportation (ground, air, or even water transport) depends not only on the distance but also traffic conditions (which varies according the time of the day), climatic (e.g., rain, snow, storms), and geographic barriers (e.g., mountain, rivers, sea) as well as the need for critical care support during transport and the cost-benefit of the different options.

In the same direction, it has also become critical to consider the degree of efficiency across the different PSCs and CSCs by carefully tracking key workflow and performance metrics including (but not limited to) their DTN and door-to-reperfusion times. For instance, in the regional scenario where a highly efficient CSC has DTN times 20 min shorter than the closest PSC, a direct transfer to the CSC might be the preferable approach even if that means that bypassing the PSC would result in a transportation time that is 15 min longer. In cases of high probability of LVO, even longer transportation delays could be justifiable. In contrast, if a PSC has short DTN time and CSC has long DTN and door-to-reperfusion times, the DS model may be favored.

Notably, there are five ongoing randomized trials comparing primary EVT vs. bridging therapy for patients directly presenting to thrombectomy capable centers. These include the SWIFT-DIRECT trial in Europe and Canada, the DIRECTSAFE trial in Australia, the SKIP in Japan, the DIRECT-MT trial in China, and the MRCLEAN No-IV trial in the Netherlands. These trials will provide critical insights about the differences in safety, efficacy, and cost-effectiveness across these two approaches and their results may lead to significant changes in the current field triage algorithms.

At the moment, there is no evidence to address the question of which patient transfer option is ideal e.g., MS or DS. The RACECAT trial is a prospective, multicenter, cluster randomized study in Catalonia territory that aims to evaluate whether a pre-hospital triage system to determine either a nearest rt-PA-capable hospital or bypass the closest facility to bring the patient to one that offers EVT (DS vs. MS), increases the efficiency of reperfusion treatments and leads to better long-term functional outcomes. Results of RACECAT will provide important answers to many of our current dilemmas (30). In addition, PRESTO (Pre-hospital Routage of acute Stroke Patients with Suspected Large Vessel Occlusion), is a French phase III randomized trial that will compare mother-ship vs. drip-and-ship strategies in terms of QUALYs (31). The results of these trials will provide important answers to many of our current dilemmas. While, stroke networks share similar issues, the results will have to be thoughtfully studied, compared, and adapted before full implementation in other stroke networks as many do not share the same organization, financial, and geographical conditions.

In each regional network, we can identify variables that favors MS or DS transfer models, so customization of the guideline to optimize patient selection for MS or DS will be needed and must take into account many local and regional factors, including: (1) time from stroke onset (or TLSW), (2) potential contraindications for IV rt-PA, (3) likelihood of LVO; (4) availability of PSC and CSC, (5) transport times to PSC or to CSC, (6) door in–door out times for PSC, (7) transfer time from PSC to CSC, (8) DTN (PSC and CSC) and door-to-reperfusion (CSC) times, and (9) EVT performance metrics (rates of reperfusion, favorable outcomes, symptomatic intracranial hemorrhage and other periprocedural complications, and mortality). Collaborative quality review processes involving regional EMS agencies and hospitals is therefore highly recommended for the development of operationalized bypass algorithms based on maps that consider all the aforementioned variables, according to each regional reality (32).

Applying different transportation paradigms to interventional stroke management are also possible. Other time sensitive procedures such as organ harvest have transported physicians to the patient site to improve time to procedure. Applying this same principle to interventional stroke management, lead to important time delay reductions in a proof of concept study (33).

Currently, a major challenge for pre-hospital care is the development of triage protocols to ensure that patients with a suspected stroke are rapidly identified and those with LVO profile are promptly recognized through the use of validated and standardized pre-hospital scales for stroke screening.

## Pre-hospital Scales to Detect Large Vessel Occlusion Ischemic Stroke

The public health impact of thrombectomy is highly dependent on rapid identification of severe stroke symptoms by EMS personnel and transport to a CSC with experience in providing fast, effective, and safe interventions. Despite the major therapeutic improvement, only a limited number of hospitals are EVT capable.

If a prediction instrument could reliably identify LVO in the field, those patients could be transported directly to EVT-capable hospitals, bypassing PSC and avoiding unnecessary delays. Another possibility would be that for patients with high suspicion of LVO, the same EMS team that brought the patient to the PSC could wait on site for a rapid assessment and treatment including IV rt-PA if eligible and then more promptly transfer the patient to a CSC as this would obviate the need to wait for new transportation team.

The NIHSS was found to be predictive of anterior circulation LVO strokes. For patients with <3 h from symptom onset, NIHSS scores ≥ 9 had positive predictive value of 86.4% and for patients between 3 and 6 h NIHSS scores ≥ 7 had a positive predictive value of 84.4% for LVO (34). In a recent study, of the 97 patients with suspected acute stroke (RACE score performed by EMS personnel ≥ 4) presenting to a CSC within the first 6 h from onset with NIHSS score > 10 on arrival, 11 (11.6%) had intracerebral hemorrhage and seven (7.2%) had no LVO after angiographic evaluation resulting a rate of LVO detection of 76.3% (35). Nonetheless, the NIHSS requires a greater degree of training, is too time-consuming to be performed in the pre-hospital setting and has only been validated for stroke severity assessment in the hospital environment.

As a result, it has become critical to develop objective pre-hospital triage criteria that appropriately identify patients who are most likely to benefit from services only available at CSC and therefore should be considered for direct transportation, while also facilitating the proper triage of less complex or lower acuity patients to the nearest stroke center (5, 36).

Several stroke severity scales aimed at recognition of LVO strokes in the pre-hospital setting have been published (Table 1) (37–43). They were initially derived from data sets of confirmed stroke cases or selected pre-hospital cases, and there has been only a limited number of prospective studies for their validation.

**Table 1:** Pre-hospital stroke scales and parameters assessed.

**Clinical prediction tool**	**Parameters assessed**
National Institutes of Health Stroke Scale (NIHSS)	
Cincinnati Pre-hospital Stroke Severity Scale (CPSSS) (37)	•Conjugate gaze deviation •Questions and commands •Arm weakness
Los Angeles Motor Scale (LAMS) (38)	•Facial droop •Arm drift •Grip strength
Rapid Arterial Occlusion Evaluation (RACE) (39)	•Facial palsy •Arm motor function •Leg motor function •Head and gaze deviation •Aphasia •Agnosia
3-item Stroke Scale (3-item SS) (40)	•Consciousness •Gaze and head deviation •Hemiparesis
Field Assessment Stroke Triage for Emergency Destination (FAST-ED) (41)	•Facial palsy •Arm weakness •Speech changes •Eye deviation •Extinction/neglect
Stroke Vision, Aphasia, Neglect (VAN) (42)	•Arm weakness •Visual disturbance •Aphasia •Neglect
Conveniently-Grasped Field Assessment Stroke Triage (CG-FAST) (43)	•LOC questions •Gaze •Facial palsy •Arm weakness •Speech problems

**Figure 1:**
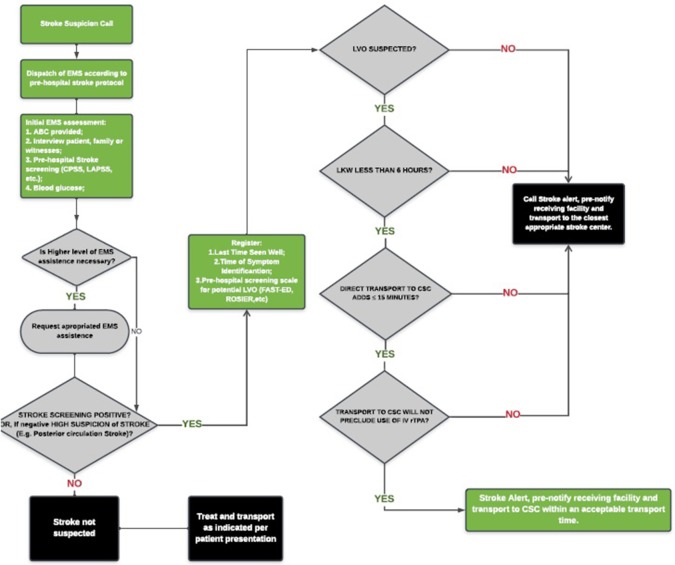
Lifeline severity-based stroke triage algorithm for emergency medical services. *Adapted from Severity-Based Stroke Triage Algorithm for EMS (44).

The performance of most available scales based on published literature was recently compared. However, at this time, there is insufficient evidence to recommend one scale over the other or a specific threshold of additional travel time for which bypassing a PSC or acute stroke-ready hospital is justifiable. The utilization of EMS stroke scales to predict LVO lack both high sensitivity and specificity, resulting on overtriage or missed cases. Moreover, it is unlikely that clinical assessment alone will have the required accuracy to diagnose LVO given the wide spectrum of symptom severity presented. Given the known impact of delays to both IV rt-PA and MT on outcome and the anticipated delays in transport for MT in eligible patients originally triaged to a PSC, the Lifeline Severity-Based Stroke Triage Algorithm was developed as an evidenced-based best-practice multi-specialty review of currently available data for EMS Stroke Triage and should be seen as broad guideline to more specific regional protocols (Figure 1) (44).

In the absence of new evidence, the Severity-Based Stroke Triage Algorithm for EMS endorses routing patients directly to CSC for clinical and transport scenarios fulfilling certain criteria. For those patients within 6 h of the time since last-known well, the algorithm favors direct transport to the nearest CSC if transport adds ≤ 15 min to transport compared with time to the nearest facility and this bypass does not preclude the use of IV rt-PA. The impact of the new trials of rt-PA beyond 4.5 h and those that address the benefit of use rt-PA or TNK prior to EVT will certainly affect the decision-making process of stroke triage. Customization of the guideline to optimize patient outcomes will be needed to account for local and regional factors, including the availability of endovascular centers, DIDO times for non-endovascular stroke centers, inter-hospital transport times, and DTN and door-to-puncture times.

## New Technologies to Aid The Pre-hospital Assessment And Decision Making

Considering the above-mentioned reasons, EMS field triage to stroke centers has gained considerable complexity. Simultaneously, system-wide implementation of transport algorithms in a stroke network is challenging. Proper selection of a destination stroke center will enhance appropriate resource utilization to meet the needs of individual patients to optimize time to reperfusion and the broader community by minimizing the time of ambulance use and distributing stroke patients more homogeneously to minimize the effects of crowding on a single healthcare system.

Although, field identification of potential candidates for MT is possible using stroke scales designed to recognize LVOS, the decision tree is substantially more complex because many of these patients are also candidates for IV rt-PA, which could often be more promptly provided at a closer location. Therefore, an optimal destination triage algorithm should not only include the probability of LVOS but also include information about the eligibility for IV rt-PA, real-time transportation time differences between CSC and PSC and the availability of human and material resources.

An ideal platform should permit customization for the needs a particular region, be cost free to the end user, and be broadly and easily available with great portability while offering a user-friendly interface and decision algorithm that decreases cognitive load. Applications designed for smartphones fulfill most of those characteristics. In fact, the use of smartphones has grown substantially among healthcare professionals and already has many different purposes in the area of stroke.

The FAST-ED (Field Assessment Stroke Triage for Emergency Destination) application is based on a built-in automated decision-making algorithm to assist EMS professionals with the decision about the most suitable destination for any given patient with acute ischemic stroke. It relies on (1) a brief series of questions assessing patient's age, anticoagulant usage, time last seen well, motor weakness, gaze deviation, aphasia, and hemineglect; (2) a database of all regional stroke centers according to their capability to provide endovascular treatment; and (3) Global Positioning System technology with real-time traffic information to compute the patient's eligibility for rt-PA or EVT as well as the distances/transportation times to the different neighboring stroke centers (45).

Effective communication is a highly valued attribute in a stroke network. Join (Allm Inc., Japan) is an application designed to simplify sharing of clinical and radiological medical information as well as to provide a platform for active communication. It offers a wide range of usages including: messaging, group chatting, integration with PACS system, streaming of live feed videos, and time tracking. It is also possible to provide integration with other applications such as the FAST-ED app thus providing support not only to effective communication but also to shared decision in complex situations.

Considering the limitations of pre-hospital scales for diagnosing LVO strokes given the wide spectrum of symptom severity, new technologies have been proposed and developed to increase triage accuracy. Transcranial Doppler (TCD) is a strong candidate for triage evaluation of LVO since is portable, non-invasive, has low cost and have been validated. However, it needs proper training and specialized personnel for reliably results. The Lucid Robotic System (Lucid M1 Transcranial Doppler Ultrasound System, Neural Analytics Inc., USA) is a robotically assisted ultrasound system for brain health assessment. It relies on evaluating the cerebral blood flow velocity (CBFV) morphology to compute a quantitative diagnostic metric called the Velocity curvature index (VCI). VCI has shown similar accuracy to the others specific TCD findings with LVO, and a recent study showed superiority of VCI over a standard Velocity Asymmetry Index (VAI) with accuracy of 88% and 79% to detect LVO, respectively. The SONAS (BURL Concepts Inc., USA) is a portable battery-powered TCD that utilizes ultrasound microbubbles as acoustic traces to detect LVO by an algorithm that evaluates brain perfusion semi-quantitatively, but it results have not been published yet.

An attractive strategy to expedite treatment and improve outcomes is the concept of “bringing the hospital to the patients.” This strategy is based on the use of ambulances (mobile stroke units) equipped with imaging system (including CTA and CTP as new development), point-of-care laboratory, telemedicine connection, and appropriate medication. Studies of pre-hospital stroke treatment have consistently shown a reduction in delays before thrombolysis and in to the triage to the appropriate target hospital (e.g., primary vs. CSC) (3, 11, 22)

The volumetric impedance phase shift spectroscopy (VIPS) is a non-invasive device (Cerebrotech Medical Systems, Pleasanton, California, USA) that aims to detect hemispheric bioimpedance asymmetry. The fluid and electrolyte changes caused by brain ischemia produces alterations on electrical properties that modifies the cerebral bioimpedance signature on the affected hemisphere allowing the detection of severe strokes, including LVO, with a sensitivity of 93% and specificity of 92% although it does not separate out ischemic from hemorrhagic strokes.

New technologies for pre-hospital assessment including identification and proper triage of LVO is a fast-evolving field, that will definitively influence decision-making process and organization of stroke networks. Nonetheless, their usefulness is yet to be tested in RCT and real-life scenarios.

As new data comes into play, the pre-hospital assessment and management of acute stroke will continue to evolve, changing the way decisions are made nowadays. New technologies will have increasing influence either in improving diagnostic accuracy in the prediction of LVO strokes, in improving communication across stroke networks as well as aiding health care professionals in the multi-factorial decision-making process.

## Author Contributions

FL and RN conceived and wrote the manuscript. FM and DB made critical reviews of the manuscript.

### Conflict of Interest Statement

The authors declare that the research was conducted in the absence of any commercial or financial relationships that could be construed as a potential conflict of interest.
